# Bacterial sialoglycosidases in Virulence and Pathogenesis

**DOI:** 10.3390/pathogens8010039

**Published:** 2019-03-24

**Authors:** Preethi Sudhakara, Iyappan Sellamuthu, A. Wilson Aruni

**Affiliations:** 1 Department of Genetic Engineering, SRM Institute of Science and Technology, Chennai 603203, India; miyupreethi@gmail.com (P.S.); iyappan.s@ktr.srmuniv.ac.in (I.S.); 2 School of Medicine, California University of Science and Medicine, San Bernardino, CA 92408, USA; 3 Musculoskeletal disease center, Loma Linda Veterans Affairs, US Department of Veteran Affairs, Loma Linda, CA 92350, USA; 4 Sathyabama Institute of Science and Technology, Chennai 600119, India

**Keywords:** sialidase, sialic acid, sialoglycoprotease, pathogenicity, therapeutic target, Siglec

## Abstract

Human oral microbiome and dysbiotic infections have been recently evidently identified. One of the major reasons for such dysbiosis is impairment of the immune system. Periodontitis is a chronic inflammatory disease affecting the tissues that surround and support the teeth. In the United States., approximately 65 million people are affected by this condition. Its occurrence is also associated with many important systemic diseases such as cardiovascular disease, rheumatoid arthritis, and Alzheimer’s disease. Among the most important etiologies of periodontitis is *Porphyromonas gingivalis,* a keystone bacterial pathogen. Keystone pathogens can orchestrate inflammatory disease by remodeling a normally benign microbiota causing imbalance between normal and pathogenic microbiota (dysbiosis). The important characteristics of *P. gingivalis* causing dysbiosis are its virulence factors which cause effective subversion of host defenses to its advantage allowing other pathogens to grow. Some of the mechanisms involved in these processes are still not well-understood. However, various microbial strategies target host sialoglycoproteins for immune dysregulation. In addition, the enzymes that break down sialoglycoproteins and sialoglycans are the “sialoglycoproteases”, resulting in exposed terminal sialic acid. This process could lead to pathogen-toll like receptor (TLR) interactions mediated through sialic acid receptor ligand mechanisms. Assessing the function of *P. gingivalis* sialoglycoproteases, could pave the way to designing carbohydrate analogues and sialic acid mimetics to serve as drug targets.

## 1. Introduction

Human microbiota consist of about 100 trilion microbial cell that constantly interact with the host counterpart through various mechanisms [1,2]. There exists a symbiotic relationship among these microorganisms, however, such a state can be reverted to exploiting pathogenic potential by certain organisms that lead to dysbiotic microbiota. Dysbiosis can lead to major pathogenic conditions especially in the gut, oral and vaginal niche, due to the richness of glycans that act as an energy source to the microorganisms. Among the host pathogen interaction strategies, many microorganisms, especially bacteria possess a strong affinity to sialic acid which coat the cell surface. Sialic acid has been predominantly found as the terminal carbohydrate in eukaryotes and prokaryotes. Sialic acid naturally occurs in prokaryotes as nine-carbon keto sugar acids derived from N-acetylneuraminic acid (Neu5Ac) [3].

The major enzyme that facilitate this interaction between the host and pathogen is bacterial “sialoglycosidases”, the enzymes that cleave the sialic acid from sialoglycoproteins. This include the sialidases and sialoglycoproteases. Sialidases (neuraminidases) are glycosylhydrolases that cleave the sialic acid (Sia) O-acceptor substrates by an exohydrolytic reaction. Functionally similar to sialidases, the O-sialoglycoprotease hydrolyzes the Sia O-acceptor substrate through an endohydrolytic reaction [4,5]. Sialidase activity has been found in viruses, bacteria, protozoa, fungi, and metazoans [5,6,7]. Bacterial sialidases have been considered virulence factors in many pathogenic organisms, such as *Corynebacterium diphtheriae*, *Vibrio cholerae*, *Streptococcus pneumoniae*, and group B *streptococci*, which colonize mucosal surfaces [8]. They have been shown to be involved in infection and tissue destruction [9], peroxide scavenging during oxidative stress [10], and the modulation of host innate immunity [5]. Furthermore, production of sialoglycosidases may be a critical factor in the provision of free sialic acid, a fermentable carbohydrate source for bacterial proliferation [8,11]. There are four mammalian sialidases namely NEU1, NEU2, NEU3 and NEU4, and they are found in lysosomes, cytosol, plasma membrane and lysosomes or mitochondria, or the endoplasmic reticulum respectively [12].

The human oral microbiome is one of the major microbiota contributing to the overall microbiome in humans. More than 700 species of bacteria with varied diversification in their composition draw interest to studying its role in both the oral and overall health of the individual. Periodontitis is a general infectious disease affecting most of the population [13]. Many commensal and pathogenic bacteria use environmental (host) sialic acids as sources of carbon, nitrogen, energy, and amino sugars for cell wall synthesis [14]. The breakdown of sialic acid residues and sialoconjugates by sialidases contributes to a wide range of important biological functions such as cellular interaction and conformational stabilization of glycoproteins in the cell membrane that could expose or mask receptors for ligand binding and other enzymatic interactions [15]. While the role of sialidase in sialic acid metabolism has been known in other oral pathogens like *Tannerella forsythia* [16], it is yet to be explored in *P. gingivalis*.

### 1.1. Sialic Acid

Sialic acid is a derivative of neuraminic acid, a monosaccharide with nine carbon acidic sugars. Sialic acids are present at the terminal location of the glycans of glycolipids, polysaccharides, and glycoproteins in the cell [17]. There are about 50 types of Sia but Neuraminic acid (Neu), N-glycolylneuraminic acid (Neu5Gc), N –acetylneuraminic acids (Neu5Ac), and deaminated neuraminic acid (KDN) are the four types of sialic acids that are the most frequent monosaccharides [17,18]. These four dominant sialic acids are subjected to variety of modifications such as substitution at, O –acetyl, O–sulfate, O-methyl, hydroxyl groups and phosphate groups [17]. Sialic acid are detected on the other surface of cells such as terminal components of glycoproteins and glycolipids and in cellular secretion of both eukaryotic and prokaryotic species [18].

A typical cell displays millions of Sialic acid molecules [19]. Given their ubiquitous presence and abundance at the surface of all cell types (including those of the immune system), Sialic acids have major biophysical effects. Earlier studies showed removal of Sialic acid from immune cell surfaces using sialidases, and showed marked changes in the behavior of such cells [20]. Removal of cell surface Sialic acids has many potentially pleiotropic effects; removal reduces the net charge and hydrophilicity of the cell surface. It can reduce the charge repulsion between adjacent cell surface molecules. It eliminates ligands for endogenous receptors like Siglecs and selectins (see below). Sialic acid removal exposes underlying glycans (mostly galactose residues), which can be recognized by other endogenous receptors, such as galectins and the galactose-binding proteins of macrophages and receptors in the neutrophils.

### 1.2. Sialidase

Sialidase (neuraminidases) are a superfamily of N-acylneuraminidase residues from the glycans of polysaccharides, glycoproteins and glycolipids that are released by glycosyl hydrolases [17]. Sialidases are found in higher eukaryotes and in some pathogenic bacteria, viruses, fungi, metazoan and protozoans. Structurally, sialidase can be divided into two types namely small and large based on the difference in molecular mass and a differential calcium requirement for protein stability or catalysis of some large sialidases [5]. The sialidases breaks down the residues of sialic acid and sialoglycoproteins that could mask or expose that receptors for enzymatic interactions and ligand binding by contributing to biological functions, such as cellular interaction and conformational stabilization of glycoproteins in the cell membranes [15].

All eukaryotes and prokaryotes exhibiting sialic acid produces sialidases but with different substrate specificities. The cleavage of sialic acid from a substrate is specific to each different sialidase. It is relied upon three important specializations that allow the eukaryotes to control their sialoglycoconjugates turnover. They are (i) the kind of kitosidic linkage, (ii) the nature of penultimate sugar residues and (iii) the type of neuraminic acid derivation [21]. But in the case of prokaryotes, the above factors do not correlate with the phylogenetic relationship [1].

## 2. Sialidase as Therapeutic Target

In the human genome, four sialidases homologs namely, NEU 1, NEU 2, NEU 3 and NEU 4 are identified. All these enzymes have different substrate specificities [22,23]. Some of the examples of sialidases as therapeutic targets are given below.

Sialidase activity on cancer cells shows that all four types of sialidase homologs behave in different manners in carcinogenesis [24]. Out of these four, NEU 1, NEU 2, NEU 4 shows a down regulation with suppression of metastasis and tumor progression [25,26], while NEU 3 shows a tendency of up – regulation [26]. Further pathological studies of NEU 3 will help to know its application in control of cancer cells [22].

Epilepsy is seizure disorder that causes abnormal or excessive activity of brain cells. This neurological disorder is characterized by unprovoked, recurrent seizures. It can be fatal if untreated [27,28]. There are many reasons for epilepsy being difficult to treat. One of the ways to treat the anomalous neuronal disorder is by altering the activation of sodium the channel through the negative surface charged residues of the cellular membrane from sialic acid. This method of modulating the negative surface charge of sialic acid can be a therapeutic target that can be acted upon by sialidase to treat epilepsy [29,30].

Influenza is viral infection affecting 5% of adults and 20% of children worldwide every year [31]. It targets the respiratory system affecting the brain, kidneys, heart, lungs, liver, and muscles [32]. DAS181 is a novel sialidases fusion protein which is used as a therapeutic target for influenza [33]. DAS181 inactivates the receptors of the host cells that are identified by the virus, by cleaving the sialic acid from the host cell surfaces making the host more repellent to the viruses [34,35]. Zanamivir and Oseltamivir are the competitive sialidase inhibitors which reduce the severity and duration of the illness [36].

Pneumonia is caused by *Streptococcus pneumoniae* that can cause sepsis and, meningitis after influenza infection [37]. Similar to influenza virus, *S. pneumoniae* possess sialidase activity [38]. Biofilm is important for pneumonococcal colonization. Nan A and Nan B (the genes that encode enzymes in sialic acid pathways) expressions are upregulated in biofilm formation in pneumonia and also the mutants shows a reduced ability to form biofilm thereby reducing the development and severity of pneumonia [39,40,41].

Periodontitis is a chronic oral inflammatory disease that initially causes infection in the tooth pockets, eventually leading to tooth loss [42]. Two major pathogens that causes periodontitis are *Porphyromonas gingivalis* and *Tannerella forsythia* [43]. *P. gingivalis* and *T. Forsythia* possess neuraminidase activity that inhibits the biofilm formation however, the pathological role of the sialic acid enzyme is yet to be discovered [44,45]. Apart from the above disease, sialidase is used as a therapeutic agent in bacterial vaginosis [46,47,48], in cystic fibrosis [49,50,51], Chagas disease [52,53] and in other bacterial and viral diseases. Therefore, sialidases are at an infancy level in terms of being used as a therapeutic target.

## 3. Oral Sialidase

In prokaryotes, over 70 different micro-organisms have been reported with sialidase activity [21]. When bacterial sialidases comes in contact with mammalian host, they become commensals or a pathobiont [17]. During the protein secretion process, the secretory proteins which are bacterial sialidases containing single peptides are cleaved [2]. The optimum pH for the monomeric bacterial sialidases ranges from 5–7 and their molecular weight ranges from 40–150 KDa [17,19,20,54]. Oral bacteria that expresse sialidase degrade sialoglycoprotein substrates. They use sialic acid as sugars to improve its growth [55]. Oral viridans Streptococci, inclusive of *Streptococcus oralis*, *S.intermedine*, *S.pneumoniae*, and *S. mitis* strains produce sialidases [56,57]. In the case of Streptococcus strains, sialic acid (Neu5Ac) is mostly used as a source for carbon [57]. Most notably red complex organism *Tannerella forsythia* exihibits sialidase dependent growth in biofilm and also produces inhibitors that might be used as adjuncts in periodontal therapy [58]. Sialidases play a pivotal role in the virulence and pathogenesis of the bacteria owing to their microenvironment being rich in mucin and the prevalence of other sialoglycoprotein rich environment such as the saliva. Certain bacteria has gene machinery that are involved in metabolism of sialic acid however, some other bacteria do resort to alternative pathway of sialic acid metabolism.

## 4. Porphyromonas gingivalis Sialidase

*Porphyromonas gingivalis* is a non-motile, asaccharolytic, Gram–negative anaerobe that plays a vital role in chronic periodontitis [42]. This bacterium mostly depends upon the energy produced by the amino acid fermentation to survive in periodontal pockets [59]. It has been proven in studies that *P. gingivalis* is associated with certain major systemic diseases such as cardiovascular disease, preterm birth and diabetes [60].

We identified that three sialidase related genes in *P. gingivalis* have shown a specific pattern of clustering with other associated genes from the bacteria [61]. Enzymes such as sialidase and sialoglycoprotease are the key factor in satisfying the asaccharolytic requirements of *P. gingivalis* by breaking down the glycoprotein conjugate; role of these enzymes in sialic acid metabolism and its involvement in protein stability, inclusive of gingipains. Hence it is believed that the absence or presence of sialic acid modulates the important proteins that are involved in both pathogenicity and metabolism of the organisms. It also implied that synergy between sialidases and sialoglycoproteases are required by *P. gingivalis* to colonize the periodontal pockets [61].

Parker, et al. [39], Roy, et al [16] and Soong, et al. [62] have proven that several bacteria, including *Tannerella forsythia,* exhibit biofilm production. In case of *P. gingivalis*, the invasion rates were significantly higher than the wild type compared to the mutant. Hence, the role of sialidases and sialoglycoproteases are unclear in *P. gingivalis*. While the role of sialidases in other oral pathogens are known, the roles of *P. gingivalis*’s sialoglycosidases in virulence modulation and pathogenesis is yet to be explored [61].

## 5. Immune Evasion and Host Sialic Acid Interaction in Pathogenesis

Sialidases is one of many hydrolases which is related with the host bacterial invasion. The initial step in sialoglycolconjugate degradation starts with the action of sialidases due to the non – reducing terminal position of sialic acid residues in oligosaccharides [8].

Sialic acid metabolism by bacteria starts by secreting a large amount of sialidases with high specific activity which is inducible [63,64]. This role is associated with both pathogenic and nutritional roles performed by the bacterial sialidases [65]. Sialic acid permease is a specific transporter for sialic acid which utilizes carbon source as an energy to transport sialic acid for degradation of cellular sialic acid [66,67,68,69]. This degradation is attained by the action of sialidases on sialo – glycoprotein releasing sialic acid (Neu5Ac) through the sialic acid permease, leading to degradation of Neu5Ac to N – acetylmannosamine (ManNAc) by an enzyme called acylneuraminate pyruvate lyase. This enzyme is induced by sialic acid and it is cell bound [1,68,70]. The end point N –acetylmannosamine (ManNAc) is apparently the central intermediate as it can be either used or broken down in the biosynthesis of sialic acid [71]. The activity of sialidases is closely associated with the essential needs for the metabolism of a bacteria [5,66,67,72].

Bacterial sialidases are considered to be virulence factors that aids in invasion and also spread of the micro-organism into the host [54,70]. The sialidases have a direct toxic effect on the host tissues and other defensive metabolisms [8].

Thus, the general characteristics of sialidases and sialic acid metabolism in immune evasion and host pathogenesis are as follows [8]:

- Large amount of extracellular enzymes with high specific activity;
- Inducible activity at the site of infection;
- Specificity of substrate shown at the site of colonization.

## 6. Siglecs

Siglecs are sialic acid binding Immunoglobin (Ig) like lectins. They are the proteins that are present on the cells of the immune system that help in binding sialic acid [73,74]. Based on their similarity in sequence, Siglecs is divided into two subsets. Sialoadhesin (CD169; Siglec 1); CD22 (Siglec 2), Myelin–associated glycoprotein (MAG; Siglec 4) and Siglec 15 are distantly related by ~25–30% sequence identity. CD33 (Siglec 3) is the main subset of Siglec which shares ~50–99% sequence identity, and is progressing promptly among mammalian species [75,76]. Immunoreceptor tyrosine – based inhibitory motifs (ITIMs) are one of the signaling motifs based on tyrosine that are present in Siglecs are involved in cell signaling and Siglec endocytosis [77].

Several Siglecs that interact with specific sialic acid modifications are expressed in human pathogens. Siglec–dependent recognition of human pathogen glycans leads to either advantage or detriment of pathogens that can alter the immune responses [75]. Phagocytosis could feasibly be inhibited by structural modifications of Toll like receptors (TLRs), Pathogen recognition receptors (PRRs) and complements leading to various immune signaling events in neutrophils. This process establishes chronic periodontitis due to a transition from microbial homeostasis to dysbiosis [78]. One of the immune mediators involved in such host inhibitory signaling is Siglec-9. Siglec-9 interaction with sialophorin (CD43), a surface sialoglycoprotein is selectively expressed on lympho-hemopoietic cells [79]. *P. gingivalis* sialoglycoproteases expose sialic acid and modify sialoglycans on *P. gingivalis* virulence factors causing the following effects: (i) the exposed sialic acid interacts with Siglec-9 via the sialic acid binding ligand (sialophorin–CD43), attenuating the host inflammatory signaling in neutrophils, and (ii) the modified sialoglycans evade Siglec-1-mediated phagocytosis because of a change in conformation, masking the sialic acid, and preventing Siglec-1 interaction (personal communication)

Innate immunity is the first line of body defense that consists of cellular and humoral immune components. Siglec acts as a negative regulator in immune cells by inhibiting the immensity of immune responses [77,80,81]. Normally, humans lack N-glycolylneuraminic acid (Neu5Gc) because of the Cytidine monophosphate – N –acetylneuraminic acid (CMAH) gene mutation; which encodes for the enzyme that is needed to convert Neu5Ac to Neu5Gc [82]. Siglec (sialic acid binding Immunoglobin (Ig) like lectin) are greatly influenced by sialic acid as it binds to sialylated glycan. The resulting glycolipids and glycoprotein have the prospective to function for Siglecs and other glycan–binding proteins as counter receptors [83].

## 7. *P. gingivalis* sialoglycosidases

### 7.1. P. gingivalis and Dysbiosis

*P. gingivalis* is an important keystone periodontal pathogen also implicated in systemic infections [15,64]. This anaerobic bacteria interferes with host immunity, enabling the emergence of dysbiotic communities [5,15]. *P. gingivalis* causes dysbiosis by subverting host defenses to its advantage [1], but the mechanisms leading to dysbiosis are poorly understood. Polymorphonuclear leukocytes (neutrophils) represent the primary cellular defense system in healthy oral tissues [19]. Neutrophils are the most common leukocytes recruited to the periodontal pocket and are needed for tissue homeostasis [65]. Recent studies indicate that neutrophils can assist the initiation and progression of periodontitis when their function is subverted by periodontal bacteria [20]. Hence, neutrophil-*P. gingivalis* interactions and subversion of innate immunity are key contributing factors to the pathogenesis of periodontal disease.

### 7.2. Mechanisms of Neutrophil Subversion and Gap

To date, most studies on *P. gingivalis* neutrophil subversion have focused on integrins and complement mediated processes [1]. Both hypo- and hyper-recruitment of neutrophils can occur due to deficiencies in the expression of β2 integrin or their negative regulators, respectively; either scenario causes unwarranted IL-17-dependent inflammatory bone loss. Moreover, microbial hijacking of C5aR (CD88) signaling in neutrophils impairs neutrophil antimicrobial function while promoting destructive inflammatory responses [20,65]. While neutrophil homeostasis plays an important role in periodontitis, the role of sialic acid mediated mechanisms causing neutrophil subversion has not been studied in *P. gingivalis*. A variety of important Siglec (sialic acid recognizing immunoglobulin-like receptor) interactions with bacterial, viral, and protozoan pathogens are beginning to be recognized. Recent research has shown that pathogenic group B *Streptococci* (GBS) binds to these Siglecs in a sialic acid-dependent fashion to downregulate leukocyte bactericidal capacity [66].

### 7.3. Sialic Acid Interactions in Virulence and Immune Interactions

Though earlier studies on many human commensal bacteria focused on the role of sialic acid as a growth factor or carbon source, its unique role in virulence and immune subversion is yet to be explored. *P. gingivalis* relies on interactions with the host sialoglycoproteins to mediate several virulence and pathogenesis factors, including adhesion, internalization and manipulation of innate and adaptive immunity [67], because the breakdown of sialoglycoproteins and sialoglycans exposes terminal sialic acid. The mechanism of immune regulation through sialic acid interactions in *P. gingivalis* has not been explored.

### 7.4. P. gingivalis sialoglycoproteases

A number of bacterial proteases have been associated with virulence; sialoglycoproteases are unique and ubiquitous in the bacterial kingdom, and have been studied in the context of virulence to some extent. However, they have not been studied for their role in immune modulation. The periodontal pocket is a rich source of sialoglycoproteins, which are found in saliva and gingival crevicular fluid [68]. The enzyme “sialoglycoproteases” expose sialic acid by breaking down sialoglycoproteins [42,56,57,58,59,60]. Among the red complex bacteria, only the *P. gingivalis* genome codes for two sialoglycoprotease genes. The role of oral bacterial sialoglycoproteases in host pathogen interactions has not been explored. Among the few other groups that study sialic acid function, we are the only group to study *P. gingivalis* sialoglycoproteases and have the lead in this area.

### 7.5. Sialic Acid Specific Interactions in Neutrophils

Phagocytosis could feasibly be inhibited by structural modifications of TLRs, PRRs and complements, leading to various immune signaling events in neutrophils. This process establishes chronic infection due to a transition from microbial homeostasis to dysbiosis [78]. One of the immune mediators involved in such host inhibitory signaling is Siglec-9. Siglec-9 interacts with sialophorin (CD43) a surface sialoglycoprotein thst is selectively expressed on lympho-hemopoietic cells [79]. One of our studies hypothesize that *P. gingivalis* sialoglycoptoeases both expose sialic acid and modify sialoglycans on *P. gingivalis* virulence factors causing the following effects: (i) the exposed sialic acid interacts with Siglec-9 via the sialic acid binding ligand (sialophorin-CD43), attenuating the host inflammatory signaling in neutrophils, and (ii) the modified sialoglycans evade Siglec-1-mediated phagocytosis because of a change in conformation, masking the sialic acid, and preventing Siglec-1 interaction.

### 7.6. P. gingivalis sialoglycoproteases in Virulence Modulation

Most interactions between bacterial pathogens and their hosts are influenced by the pattern of expressed glycans and glycan-binding receptors (lectins, adhesins or agglutinins) [66]. Several medically important bacterial pathogens display sialic acid on their surface as an anti-recognition molecule; this allows bacteria to masquerade as “self” and thereby elude or subvert host immune responses. Exploring the mechanisms by which sialylated pathogens exploit host receptor systems to modulate virulence is novel and new. We have identified unique sialoglycans on the surface of the *P. gingivalis* wild type (W83) cell surface that were missing in PG-Sgps isogenic mutants. Capsular polysaccharides of pathogenic bacteria such as Group B *Streptococci* display sialoglycan structures that both resemble vertebrate glycoproteins and can bind to Siglecs on leukocytes [69]. Our preliminary data showed that *P. gingivalis* exhibits a similar sialoglycan structure, with sialic acid linkages [39]. Also, certain Siglecs, such as Siglec-9 on neutrophils bind to host α2,3 linked sialic acid, causing inhibitory signals. We have shown that *P. gingivalis* interacts with α2,3 and α2,6-linked sialic acid moieties in human cells. The capsular glycan of *P. gingivalis* can elicit the inflammatory cytokines MIP-2, RANTES and MCP-1 in murine macrophages. This suggests interactions in both neutrophil adhesion and host inhibitory signaling, which are two key mechanisms utilized by the bacteria.

## 8. Conclusions

Bacterial sialidases and their sialoglycan targets contribute to host–microbe interactions at the mucosal surface. Such mechanisms play a pivotal role in both the oral and gut microbiome where there is rich mucin environment and profuse sialoglycoproteins in the saliva and in the gut mucus respectively. An imbalance in the proportion of gut commensals able to modulate mucosal sialic acid levels or a change in host mucin sialylation is often associated with enteric infection or intestinal inflammation. Maintaining a balance in the ability of commensals to produce or consume sialic acid in the mucosal compartment is therefore essential to oral and gut homoeostasis.

Further investigations of bacterial sialidases should clarify the type of sialylated structures that are accessible to the bacteria and the specificity of sialidases towards sialic acids with different modifications and in different linkages. These include gaining structural insights into the diversity of sialic acid derivatives that can be produced or taken up by commensal and pathogenic bacteria. Thus, for therapeutic purposes, modulation of sialidase expression might be effectively achieved by appropriate use of specific inhibitors or pro – or prebiotic approaches targeting specific bacterial strains. This approach will play a role in reverting dysbiotic infections.
